# Pyroptosis in Liver Disease: New Insights into Disease Mechanisms

**DOI:** 10.14336/AD.2019.0116

**Published:** 2019-10-01

**Authors:** Jiali Wu, Su Lin, Bo Wan, Bharat Velani, Yueyong Zhu

**Affiliations:** ^1^Liver research center of the First Affiliated Hospital of Fujian Medical University, Fujian 350005, China; ^2^Faculty of Life Sciences and Medicine, King’s College London, London SE1 1UL, United Kingdom; ^3^Basildon and Thurrock University Hospitals NHS Foundation Trust, Nethermayne, Basildon, Essex SS16 5NL, United Kingdom

**Keywords:** pyroptosis, NLRP3, fatty liver disease, liver failure

## Abstract

There has been increasing interest in pyroptosis as a novel form of pro-inflammatory programmed cell death. The mechanism of pyroptosis is significantly different from other forms of cell death in its morphological and biochemical features. Pyroptosis is characterized by the activation of two different types of caspase enzymes—caspase-1 and caspase-4/5/11, and by the occurrence of a proinflammatory cytokine cascade and an immune response. Pyroptosis participates in the immune defense mechanisms against intracellular bacterial infections. On the other hand, excessive inflammasome activation can induce sterile inflammation and eventually cause some diseases, such as acute or chronic hepatitis and liver fibrosis. The mechanism and biological significance of this novel form of cell death in different liver diseases will be evaluated in this review. Specifically, we will focus on the role of pyroptosis in alcoholic and non-alcoholic fatty liver disease, as well as in liver failure. Finally, the therapeutic implications of pyroptosis in liver diseases will be discussed.

Programmed cell death (PCD) contributes to the development of liver disease [1, 2]. Recently, pyroptosis, a novel kind of PCD, was proven to play an important role in liver diseases. Pyroptosis was first described and named in 1992 by Zychlinsky[3], and was characterized by caspase-dependent (caspase-1/4/5/11) pore formation in the cell membrane and subsequent release of pro-inflammatory mediators(interleukin-18/1β, IL-18/1β). Cell swelling, hyperpermeabilization of the plasma membrane, rapid cell lysis and release of cytoplasmic content and pro-inflammatory mediators are distinguishing features of pyroptosis [4].

Pyroptosis is an innate immune defense against intracellular bacteria[5]. However, increasing evidence indicates that it also participates in sterile inflammation, such as in acute or chronic liver diseases. Research on the molecular mechanism of pyroptosis will contribute to a better understanding and management of liver diseases. In this review, we will first describe the mechanism and biological significance of pyroptosis, including the initiating events, receptors, signaling pathways, biological cellular outcomes and the downstream effects of pyroptosis. Then, we will summarize the evidence from the latest basic and clinical research regarding pyroptosis in the field of hepatology. The therapeutic implications of pyroptosis in liver diseases will be discussed at the end.

## 1. Differences between pyroptosis, necrosis and apoptosis

Apoptosis and necrosis are common types of PCD found in the liver[6]. Morphologically similar to necrosis, pyroptosis can lead to membrane rupture and pore formation[7]. In contrast to pyroptosis, necrosis is characterized by mitochondrial impairment, depletion of adenosine triphosphate (ATP), and failure of ATP-dependent ion pumps. Necrosis is mainly caused by physical and chemical stimulation and leads to cell membrane rupture and release of cytoplasmic contents that eventually results in inflammation[8].

Apoptosis is mechanically similar to pyroptosis as they are both triggered by caspases. The caspases in apoptosis can be stratified into initiator caspases (caspase-2/8/9/10) and effector caspases (caspase-3/6/7)[9]; in pyroptosis, the caspases are caspase-1/4/5/11[4, 10, 11]. Apoptosis can be initiated by death signals through the intrinsic pathway(cellular stresses) or extrinsic pathway[12]. The B-cell lymphoma 2 (Bcl-2) family members and the tumor necrosis factor (TNF) family members are prominent regulators of apoptosis[13, 14]. In pyroptosis, the inflammasome and some danger signals are activators of caspases. Morphologically distinct from pyroptosis, apoptosis is characterized by cellular shrinkage, nuclear condensation, and fragmentation. Cellular material is not released from the cell and inflammatory cytokines are not produced during this process[9, 12].

The differences in the morphological and molecular pathways between the different forms of cell death are listed in Table 1

**Table 1:** Comparison of different forms of cell death

Cell death	Activated by	Effector	Morphology	Result cell corpse	inflammation	refs
Pyroptosis	PAMPs and DAMPs	caspase-1 or caspase-4/5/11	lytic	pore-induced intracellular trap	yes	[4] [10][11]
necrosis	physical and chemical stimulation	-	lytic	pore-induced intracellular trap	yes	[8]
apoptosis	intrinsic or extrinsic pathways	caspase-3/6/7	non-lytic	apoptotic body	no	[9] [12]

PAMPs: pathogen-associated molecular patterns; DAMPs: Damage-associated molecular pattern molecules

## 2. Biological significance of pyroptosis

Pyroptosis is an effective immune defense against intracellular bacterial infection [15-19]. Pyroptosis begins with recognition of pathogen proteins, continues with cleavage of the pyroptotic substrate gasdermin D (GSDMD) and finally ends with formation of pore-induced intracellular traps on the host cell membrane [20]. Through this mechanism, pyroptosis helps to capture and clear pathogens by recruiting neutrophils to the infection site. Additionally, pathogens can also be eliminated by secondary insults, for instance, hydrogen peroxide[5, 21]. Inflammatory cytokines and cellular contents flow out of the cell after pore formation and cell lysis, resulting in the pro-inflammatory cascade. This immune response against pathogens can be beneficial in the clearance of infectious organisms. However, excessive host cell pyroptosis is harmful to healthy tissue if not well regulated. A well-known example of uncontrolled immune response to pathogens is sepsis [22]. In this way, pyroptosis is a double-edged sword.

In general, the balance between chronic inflammatory injury and the healthy immune response of pyroptosis is precisely regulated. When the balance is disrupted, excessive host immune response and massive cell death during pyroptosis can lead to serious disease. Inflammasome activation, which occurs at the onset of pyroptosis, is mechanically believed to be involved in the development and progression of the following diseases: Alzheimer's disease [23], systemic lupus erythematosus [24], cataracts [25], liver diseases [26-28], renal ischemia reperfusion injury [29] and diabetes [30]. Additionally, cancer development is associated with pyroptosis [31]. As a result of pyroptosis, the release of IL-18/IL-1β and change in innate immunity provide the pro-inflammatory microenvironment necessary for tumor development [32].

## 3. Molecular mechanism of pyroptosis

### 3.1 Initiating of the pyroptosis

**Figure 1.**
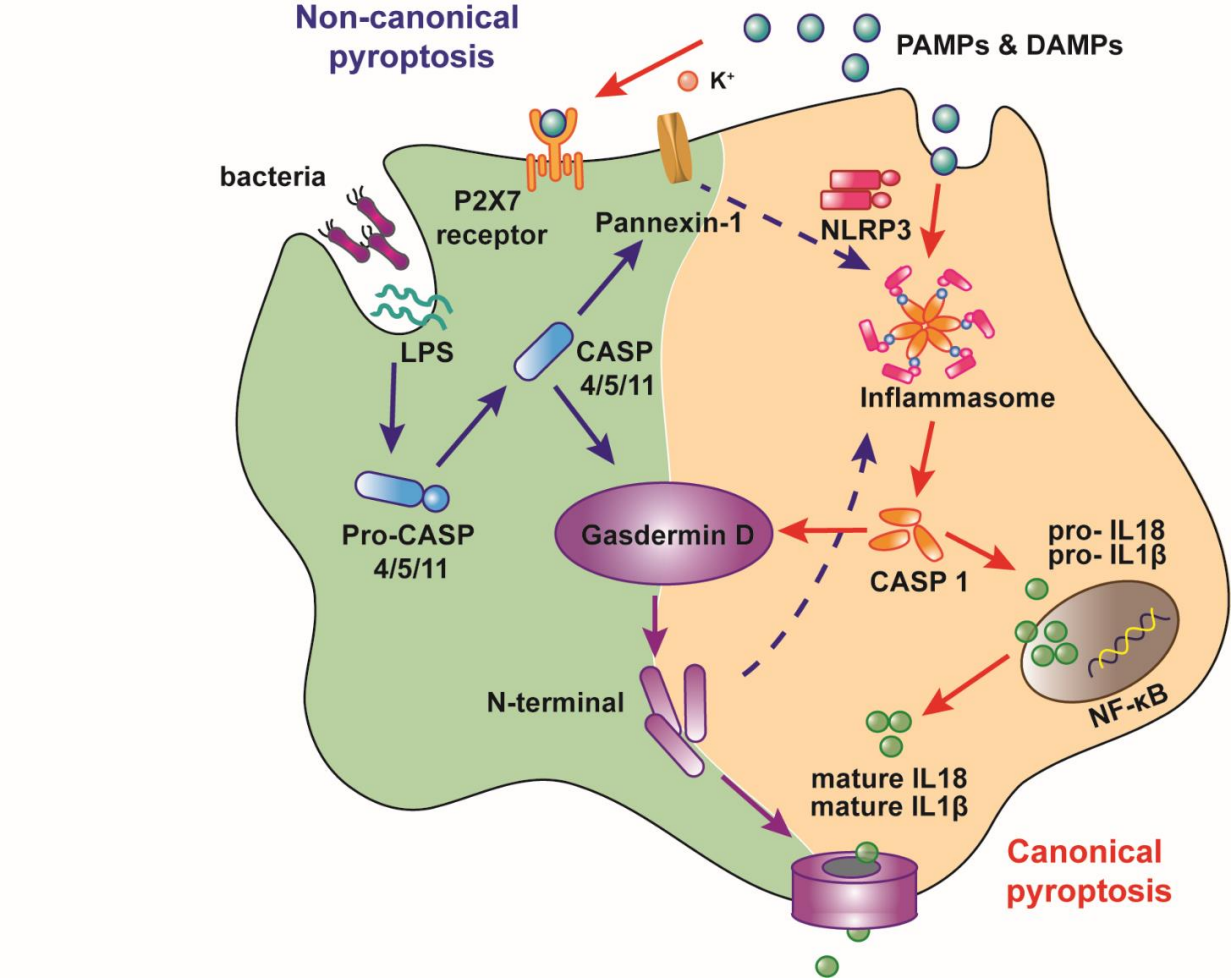
Pathways of pyroptosis. There are two different pyroptotic pathways. The canonical pyroptosis is dependent on the activation of caspase-1 by inflammasomes, which can recognize PAMPs and DAMPs. Compared to canonical pyroptosis, noncanonical pyroptosis is mediated by the activation of caspase-1 and caspase-4/5 (caspase-11 in mice), which can be directly activated by LPS independent of TLR4. Upon activation, these caspases cleave gasdermin D then bind to lipids in the plasma membrane and form oligomeric pores leading to the release of cellular contents and cell death. Caspase-4/5/11 activates the Pannexin-1 channel and then opens the P2X7 pore to mediate pyroptosis. Meanwhile, activation of caspase-1 results in the cleavage of pro-IL-1β and pro-IL-18 and the production of mature cytokines. PAMPS, pathogen-associated molecular patterns; DAMPS, damage-associated molecular patterns; IL-1β, Interleukin-1β; IL-18, Interleukin-18.

There are two different pyroptosis pathways (Fig. 1): canonical pyroptosis, which is dependent on caspase-1 activation [10], and noncanonical pyroptosis, which is dependent on caspase-4/5/11 activation[11]. Canonical pyroptosis starts with inflammasomes recognizing various exogenous and endogenous danger signals, including pathogen-associated molecular patterns (PAMPs) and damage-associated molecular pattern molecules (DAMPs), and caspase-1 is subsequently activated[10]; noncanonical pyroptosis is dependent on caspase-4/5 (the caspase-11 in mice), which can be directly activated by lipopolysaccharide (LPS) independent of Toll-like receptor 4 (TLR4)[33].

### 3.2 Receptors of pyroptosis

Stimulatory signals are received by inflammasomes in canonical pyroptosis. Inflammasomes are intracellular, multiprotein complexes that usually consist of three parts: a cytosolic sensor, a bridging adaptor and an effector. The role of inflammasome activation in liver diseases has been extensively reviewed by Szabo and Petrasek [27].

The sensor component of the inflammasome system is formed by nucleotide-binding oligomerization domain (NOD)-like receptors (NLRs). According to different cytosolic pattern recognition receptor (PRR) proteins, NLRs can be categorized as NLRP1, NLRP3, NAIPs (NLR family apoptosis inhibitory proteins), NLRC4, or AIM2 (absent in melanoma-2)[34-36].

Apoptosis-associated speck-like protein containing caspase recruitment domains (ASCs) are the bridge adaptor of the inflammatory complexes. It contains two domains: one is the pyrin domain (PYD) which interacts with the PRR of the sensor, and the other is the caspase-recruitment domain (CARD) which interacts with pro-caspase-1 effector. Through these two domains (pyrin and CARD), ASC can interact with the cell death executioner and act as an essential adapter for inflammasome integrity [37, 38].

The effector in canonical pyroptosis is pro-caspase-1[10]. Although caspase-1 is commonly activated by the canonical mechanism, there are two noteworthy exceptions. The first involves the NAIP-NLRC4 and NLRP1b molecules. These two molecules lack a PYD domain and can directly recruit a bridging ASC to activate pro-caspase-1 without inflammasome complex formation or autoproteolysis [39, 40].

In noncanonical pyroptosis, pro-caspase-4/5 (caspase-11 in mice) is the sensor and can be directly activated by LPS independent of TLR4[11, 41]. The lipid-A portion of LPS binds to the CARD domains of these inflammatory caspases (4/5/11) and then promotes their oligomerization and activation, which eventually leads to the cleavage of GSDMD and pyroptosis[42].

### 3.3 Signaling pathways

The initiation of pyroptosis begins with the aforementioned recognition of PAMPs and DAMPs. The receptors help to translate extracellular signals into cells. The subsequent activation of caspases is the key step in pyroptosis signaling pathways.

Caspase-1 is the only reported caspase involved in canonical pyroptosis. The activation of caspase-1 by the NLRP3 inflammasome leads to cleavage of pro-IL-1β and pro-IL-18 and the production of mature, biologically active cytokines [10, 35]. Additionally, activated caspase-1 also cleaves the pyroptotic substrate GSDMD into a N-terminal fragment and a C-terminal fragment. The oligomerized gasdermin-N forms the membrane pores and induces pyroptosis [43-45]. The GSDMD pores in the membrane also allow the release of IL-1β and IL-18 and disperse soluble cytosolic contents including the intracellular enzyme lactate dehydrogenase (LDH) [44, 46, 47].

In noncanonical pyroptosis, caspase-4/5/11 cleaves and activates GSDMD. This process is similar to canonical pyroptosis. In addition, caspase-1 is activated by cleaved GSDMD through the combination of NLRP3 and ASC[48]. Caspase-4/5/11 can also activate pannexin-1, subsequently open the membrane channel P2X7, and eventually cause formation of small pores in the cell membrane[49]. On the other hand, activated pannexin-1 can trigger the NLRP3 inflammasome through K^+^ efflux and ultimately leads to IL-1β production and release [49]. Through the GSDMD and pannexin-1, noncanonical and canonical pyroptosis are linked together.

In addition to GSDMD, other members of the gasdermin family also participate in pyroptosis. A recent study showed that gasdermin B (GSDMB) promotes pyroptosis^[50]^. Unlike GSDMD, the N-terminus of GSDMB does not directly induce cell death. It binds to the CARD domain of caspase-4, which is required for the cleavage of GSDMD in noncanonical pyroptosis, and activates it [50]. Genome-wide association studies revealed a correlation between GSDMB gene polymorphisms and increased susceptibility to Crohn’s disease [51] and asthma [52]. Another gasdermin protein, gasdermin E (GSDME), has been demonstrated to be associated with pyroptosis as well [53, 54]. Interestingly, Wang and colleagues [53] found that GSDME could switch caspase-3-mediated apoptosis to pyroptosis. However, the roles of GSDMB and GSDME in pyroptosis have not been thoroughly investigated and need further exploration.

### 3.4 Biological outcome to the cell

GSDMD pores in the cell membrane are detrimental to the cell. If the number of pores in the plasma membrane is small, a normal cellular emergency response can patch up the porous membrane [55]. However, if the number of GSDMD pores exceeds the cell’s self-repairing ability, sodium and water will enter the cell, and the cellular volume will increase beyond membrane capacity and eventually cause cell swelling and rupture.

On the other hand, the IL-1 family cytokines (IL-1β/IL-18) that are released through the GSDMD pore during pyroptosis will recruit immune cells to the site of inflammation, stimulate secondary cytokine production and trigger acute-phase signaling responses [56]. Beside the general effects of those cytokines on cells, IL-1β in the liver can promote proliferation and trans-differentiation of hepatic stellate cells (HSCs), the main profibrogenic cell in the liver, and cause accumulation of fibrotic tissue [57, 58].

## 4. Pyroptosis in liver diseases

### 4.1 The stimuli of pyroptosis in liver diseases

Inflammasomes can be stimulated by different kinds of substances, from PAMPs released by pathogens (bacteria, fungi and viruses) to DAMPs released by dying cells[59]. Interestingly, DAMPs from the kidney, a remote organ, also initiate pyroptosis in the liver. A recent study demonstrated that the release of extracellular histone following renal graft ischemia-reperfusion injury could mediate remote hepatic damage in rats[60]. In addition to those proteins, some chemical substances, such as mesoporous silica nanoparticles[61], benzo[a]pyrene[62] or rare-earth oxide nanoparticle (e.g., Gd2O3)[63], can activate inflammasomes in hepatic L02 cells, HL-7702 cells, and Kupffer cell(KC)/macrophages, respectively.

### 4.2 Liver cells participating in pyroptosis

The liver is the first line of defense against diverse microbial particles from circulating blood. Inflammasomes, as the receptor of pyroptosis, are highly expressed in macrophages[64], stellate cells[65], and hepatocytes[66]. Various cells in the liver participate in pyroptosis. PAMPs and DAMPs can directly induce pyroptotic death in hepatocytes or indirectly cause liver cell injury by crosstalk between cells.

#### Macrophages

There are two phenotypes of macrophages in the liver: infiltrating macrophages and resident macrophages (KCs). Both types are the first cells to detect the presence of danger signals in the liver. Pyroptosis in macrophages is responsible for the development of liver diseases. For instance, rare-earth oxide can initiate NLRP3 inflammasome and caspase-1 activation and eventually lead to pyroptosis in KCs, bone marrow-derived macrophages, and other macrophage cell lines such as J774A.1 and RAW 264.7 cells [63]. However, this phenomenon is not observed in hepatocytes [63]. In a fatty liver animal model established by a methionine choline-deficient (MCD) diet, the mtDNA released from mitochondria can activate NLRP3 inflammasomes in KC and induce IL-1β secretion[67]. Noncanonical pyroptosis in Kupffer cells can also lead to liver injury. Chen et al [68] recently demonstrated that LPS could cause release of cathepsin B and subsequently activate caspase-11 in KCs.

#### HSCs

The activation of inflammasomes in HSCs plays vital roles in the development of fibrosis[69]. Liver fibrosis is characterized by deposition of extracellular matrix, and HSCs are the primary cells responsible for extracellular matrix storage. Multiple functional changes of HSCs can be induced by the NLRP3 inflammasome, which is moderately expressed in HSCs [65, 70]. Pro-inflammatory cytokines (IL-1β and IL-18) released after NLRP3 inflammasome activation have been demonstrated to activate HSCs and tissue fibroblasts in vitro and in vivo[71, 72]. The crosstalk between cells is also involved in the activation of HSCs[70]. The cytokines and DAMPs released from injured hepatocytes and macrophages will activate HSCs and induce to liver fibrosis[65].

#### Eosinophils

Infiltration of eosinophilic leucocytes has been found in many liver diseases, including hepatic allograft rejection[73], drug-induced liver injury[74] and chronic hepatitis C[75]. A recent study showed that eosinophils also play a role in pyroptotic hepatocyte death. In Schistosoma mansoni-infected mice, S. mansoni eggs in the hepatic sinusoids can lead to liver cell death. Isolated eosinophils from the livers of infected mice display caspase-1-mediated pyroptosis[76].

#### Hepatocytes

Inflammasomes are detectable in hepatocytes. Many recent studies have uncovered the direct role of inflammasome activation in hepatocyte injury[28, 77]. In obese mice established by the MCD diet, endoplasmic reticulum (ER) stress induced by LPS challenge led to NLRP3 inflammasome assembly and subsequent hepatocyte pyroptotic death [77].

A recent study even suggested that activation of the hepatocyte-specific NLRP3 inflammasome and subsequent pyroptosis might be a more important contributor to liver injury and fibrosis than previously thought. This indirect evidence comes from Wree et al.[78], who developed the global and myeloid cell-specific Nlrp3 knock-in mice that constitutively express activated NLRP3 to elucidate differences in liver pathology when NLRP3 inflammasomes are activated in different cells. Interestingly, compared to global Nlrp3 knock-in mice, those with myeloid-specific Nlrp3 mutations lack detectable pyroptotic hepatocyte cell death and have less severe liver inflammation, HSC activation, and fibrosis. This result highlights the importance of pyroptosis in hepatocytes. In addition to immune cells, hepatocyte pyroptosis resulting from intrinsic inflammasome activation exacerbates inflammation and fibrosis in the liver, indicating that both immune cell- and liver-specific NLRP3 inflammasome activation are essential for liver injury. However, those studies did not use the hepatocytic-specific NLRP3 mutant animal, therefore more studies are needed for direct evidence of the crosstalk between hepatocyte and the other types of cells in the onset and progression of liver injuries.

## 5. Pyroptosis associated-liver diseases

### 5. 1 Nonalcoholic fatty liver disease (NAFLD)

NAFLD is characterized by lipid accumulation in the liver in the absence of significant alcohol intake, medication use or other medical conditions that cause hepatic steatosis. It can be pathologically classified into different degrees of severity, from simple steatosis to steatohepatitis (NASH) and fibrosis[79, 80]. Low-grade chronic inflammation in the liver is a generally accepted hypothesis for the underlying pathophysiology of NAFLD[81, 82]; thus, pyroptosis is considered to have an important role in the development and progression of this inflammatory disease.

Inflammasome activation in both bone marrow-derived cells and liver parenchymal cells activates caspases and promotes inflammation and fibrosis in MCD diet-induced NAFLD in mice[64]. Typical activators of inflammasomes—such as fatty acids[83], DAMPs released by dying hepatocytes and immune cells [59, 84, 85], and uric acid [86]—upregulate NLRP3 inflammasome components as described above. Moreover, pyroptosis mediated by mitochondrial dysfunction and subsequent production of reactive oxygen species (ROS) in NAFLD have drawn much attention recently[87-90]. ROS have long been considered to induce lethal hepatocyte injury in steatosis[91-93]. Injured mitochondrial-released DAMPs, including mitochondrial DNA, mitochondrial ROS and ATP[94], can promote NLRP3 inflammasome activation directly or indirectly via thioredoxin-interacting protein (TXNIP)[95] and P2X purinoceptor 7 (P2RX7)[96].

Growing evidence shows that pyroptosis is an inflammatory link between simple steatosis and NASH, as NLRP3 activation is seldom observed in an animal model of simple steatosis without inflammation[97-99]. NLRP3 activation in NASH has been shown in both human and animal models[100-102]. The pro-inflammatory cytokines released during pyroptosis are key molecules for NAFLD development. IL-1β is believed to drive the pathogenesis of liver inflammation, steatosis and fibrosis. It also has the additional effect of amplifying the response of other cytokines[103-105]. The activation of IL-1 signaling, which is downstream of inflammasomes, has been implicated in NAFLD pathogenesis[106, 107].

Pyroptosis in hepatocytes and macrophages is also involved in the development of liver fibrosis in NAFLD. After injury, these cells release DAMPs and danger signals such as IL-1β/IL-18 and inflammasome particles^[108]^. Those DAMPs and cytokines bind to receptors located on HSCs and induce upregulation of fibrotic markers, thus leading to liver fibrosis[65].

Interestingly, another cytokine cleaved by caspases during pyroptosis—mature IL-18—seems to be play a different role in NAFLD/NASH progression. In the IL-18 knock-out mouse model, the expression of gluconeogenesis genes in the liver is substantially higher. Those mice are prone to develop obesity, hyperphagia and insulin resistance[109]. It seems the pyroptosis is a coin with two sides engendering two products, one of which (IL-18) is beneficial, while the other (IL-1β) is detrimental for NAFLD. However, the role of IL-18 has not been clearly elucidated.

Although there have been many studies on pyroptosis in NAFLD, many questions remain unanswered. For example, as steatohepatitis and fibrosis only account for a small proportion of the NAFLD population[110, 111], when and why pyroptosis is initiated requires extensive investigation.

### 5.2 Alcoholic liver disease

ALD is a general term used to refer to alcohol-related liver injuries. The clinical spectrum includes steatosis, fibrosis, alcoholic hepatitis (AH), cirrhosis, and hepatocellular carcinoma (HCC)[112]. Activation of innate immunity, hepatic and systemic inflammation and macrophages is a major contributor to ALD progression[113, 114]. There is an increasing body of evidence suggesting that pyroptosis is a key driver of ALD in patients and animal models. NLRP3 deficiency prevents the development of alcohol-induced liver inflammation and has a beneficial effect on liver damage and steatosis[115]. Recently, Khanova et al.[116] uncovered upregulation of the Casp4/11 gene in liver tissue of histological-verified AH patients and mice using unbiased ribonucleic acid (RNA) sequencing analyses.

Ethanol can induce pyroptosis through different ways. One effect of ethanol on pyroptosis is via the microRNA-148a pathway. Alcohol can decrease microRNA -148a expression in hepatocytes through FoxO1 and induce the overexpression of TXNIP, a member of the α-arrestin family[117]. TXNIP then binds to NLRP3 inflammasomes and facilitates NLRP3 activation, thus leading to caspase-1-mediated pyroptosis[117, 118].

Another impact of alcohol on pyroptosis is triggered by PAMPs (derived from the gut) and DAMPs (derived from hepatocytes in liver inflammation due to alcohol exposure)[119]. The DAMPs, such as ATP and soluble uric acid, are released from damaged primary hepatocytes induced by ethanol and trigger the release of inflammasome-dependent cytokines from immune cells[115, 120]. Pyroptosis mediated by intracellular contents can stimulate and sustain the inflammatory cycle in ALD. Alcohol metabolism in hepatocytes also increases the production of ROS and leads to mitochondrial dysfunction, thus increasing the susceptibility of hepatocytes to inflammatory cytokines [121, 122].

### 5.3 Liver failure

Acute liver failure (ALF) is characterized by an abrupt onset of severe liver injury with gross hepatocyte dysfunction[123]. Acetaminophen (APAP) overdose is the most common cause of ALF. N-acetyl-p-benzoquinone imine, a reactive metabolite of APAP, is thought to directly damage hepatocytes through mitochondrial oxidative stress, c-Jun amino-terminal kinase activation, nuclear DNA fragmentation and transitions in mitochondrial permeability[124]. Additionally, inflammation derived from DAMP-mediated innate immune signaling also promotes liver injury in APAP hepatotoxicity[125]. The ATP and NAD released after death cell activates P2X7, subsequently triggers NLRP3 inflammasomes, and eventually propagates APAP-induced hepatic injury[126, 127].

Most studies of pyroptosis in liver failure are conducted in animal models. Significantly elevated levels of NLRP3, cleaved caspase-1 and IL-1β and predominant pyroptotic cell death have been observed in the livers of concanavalin (ConA)[128] and D-galactosamine (D-Gal)[129] induced liver failure models. Exposure to the NLRP3 inhibitor (MCC950) before D-Gal challenge attenuate pyroptosis injury^[129]^. TNF receptor superfamily member 4(OX40), which is expressed in liver invariant natural killer T (iNKT) cells, is also involved in the development of liver failure.OX40 activates caspase-1 via TNF receptor-associated factor 6-mediated recruitment of the paracaspase MALT1, which consequently leads to massive pyroptotic death of iNKT cells and liver injury[130]. IL-1 receptor type 1 (IL-1R1) can amplify cell death and inflammation in hepatocytes during pyroptosis in liver failure. ALF induced by D-Gal and LPS is significantly attenuated in the liver-specific IL-1R1 knock-out mice[131]. Pretreatment with the IL-1 receptor antagonist (rhIL-1Ra) strongly suppresses ConA-induced hepatitis by decreasing both TNF-α and IL-17 secretion and inflammatory cell infiltration into livers[128].

Although massive cell death and inflammation are important in the development of liver failure, the correlation between pyroptosis and liver failure has only been investigated in animal models but rarely been investigated in patients. Moreover, the role of pyroptosis has never been studied in another fatal subtype of liver failure, acute chronic liver failure (ACLF) in which systemic inflammation is believed to be a major pathogenic mechanism[132, 133]. ACLF patients have two distinguishing characteristics, chronic liver injury and acute deterioration of liver function, which complicate the elucidation of underlying mechanisms if pyroptosis is involved.

### 5.4 Viral hepatitis and pyroptosis

NLRP3 expression is upregulated in hepatocytes and macrophages with HCV[134, 135]. Previous studies showed that NLRP3 inflammasomes participated in hepatitis C[136, 137]. HCV RNA can directly induce the assembly and activation of NLRP3 inflammasomes in infected hepatocytes[137]. The secretion of IL-18 after NLRP3 activation stimulates NK cell-derived interferon-γ and thereby helps to suppress HCV[138]. The release of DAMPs from lysed pyroptotic cells can recruit immune cells and further promote secondary inflammation[137, 139, 140]. IL-1β production through the NLRP3 inflammasome in KC has been identified as the source of amplified inflammatory responses in patients with HCV infection[139].

Hepatitis B virus (HBV) is a global health problem and more than 350 million people are chronically infected. Chronic HBV infection leads to liver fibrosis, cirrhosis, liver failure and hepatocellular carcinoma; however, no cure for chronic HBV has been found to date[141]. Hepatitis B core antigen (HBcAg) is thought to be associated with pyroptosis. In 2003, Manigold et al.[142] showed HBcAg treatment increased the secretion of IL-18 in peripheral blood mononuclear cells (PBMCs) from patients with chronic hepatitis B; this effect was completely blocked by a caspase-1 inhibitor. Another interesting finding from this study was that HBcAg-induced IL-18 secretion was significantly lower in PBMCs of hepatitis B envelope antigen (HBeAg)-positive patients[142]. In 2017, Yu et al.[143] elucidated that HBeAg inhibited LPS-induced NLRP3 inflammasome activation in liver tissue of HBV-carrier mice. Using the HBV-persistent mouse model induced by hydrodynamic injection of pAAV/HBV1.2 plasmid, the researchers found that HBeAg, but not HBsAg, inhibited LPS-induced NLRP3 inflammasome activation via repression of the nuclear factor-kappa B(NF-κB) pathway and ROS production in KCs. As inflammasome activation is a pivotal immune response to pathogens, the suppression of inflammasomes by HBeAg might explain the mechanism of HBV persistence and immune tolerance. These two studies establish the possible link between pyroptosis and HBV infection; however, the role of pyroptosis in HBV infection progression is largely unknown. More studies on pyroptosis are urgently required in HBV related-diseases as they are epidemic and greatly needs a cure.

**Table 2:** Potential anti-pyroptotic targets in liver disease.

Therapeutic targets	Molecules	Diseases	Subjects	References
NLPR3	Glyburide	Acute liver injury (CLP model)	male C57BL/6 mice	[26]
	MCC950 (NLRP3 inhibitor)	ALF (D-Gal challenge)	male C57BL/6 mice	[129]
	EPO	Sepsis related liver injury (LPS challenge)	mice	[155]
	TUDCA	NASH	obese mice	[77]
	Taurine	NAFLD NASH	Male C57BL/6 mice	[156]
	Silybin	NAFLD	Male C57BL/6 mice	[153]
	Dihydroquercetin	ALD	Male C57BL/6 mice	[146]
	Chlorogenic acid	Acute liver injury (CCl4-induced)	male Sprague-Dawley rats	[154]
	Scutellarin	Sepsis related liver injury (intraperitoneally injection of Escherichia coli)	Female C57BL/6 mice	[144]
Caspases	AC-YVAD-CMK (caspase-1 inhibitor)	Acute liver injury (MSN administration)	male C57BL/6 mice	[61]
	IDN 6556 (pan-caspase inhibitor)	NASH	mice	[157]
	IDN 6556 (pan-caspase inhibitor)	HCV	HCV patients	[158]
	PF-03491390 (pan-caspase inhibitor)	HCV	HCV patients	[159]
	Cathepsin B inhibitor	SIRS /sepsis (LPS challenge)	Human KCs	[68]
IL-1	Anakinra (IL-1 inhibitor)	ALF (D-GalN/LPS administration)	mice	[131]

NLPR3: NOD-like Receptor Protein 3; CLP: Cecal Ligation and Puncture; ALF: Acute Liver Failure; D-GalN: D-galactosamine; EPO: erythropoietin; LPS: lipopolysaccharide; TUDCA: Tauroursodeoxycholic acid; NASH: non-alcoholic steatohepatitis; CTSB: cathepsin B; NAFLD: Nonalcoholic fatty liver disease; ALD: Alcoholic liver disease; CCl4: carbon tetrachloride; MSN: mesoporous silica nanoparticles ;HCV: Hepatitis C virus; SIRS: systemic inflammatory response syndrome; IL-1: Interleukin-1

### 5.5 Sepsis related liver injury

Pyroptosis has been shown to play a vital role in immune cell activation and amplification of liver inflammation in sepsis-related liver injury[26]. Sepsis is characterized by the release of several pro-inflammatory cytokines in an event commonly known as “the cytokine storm”. These cytokines, via the pyroptosis pathway, can aggravate hepatic cell death and result in liver dysfunction with a very high mortality rate. In the septic mouse model established by cecal ligation and puncture (CLP) surgery, which is a standard model for polymicrobial sepsis, hepatocyte pyroptosis increases in a time-dependent manner; this result demonstrated that the severity of liver pyroptosis is correlated with liver damage[26]. The highest hepatic cell pyroptosis rate was observed at 24 h post-operation in CLP mice. Treatment with NLRP3 and/or caspase-1 inhibitor significantly improved the survival rate and alleviated liver damage in CLP mice[26]. Inhibiting NLRP3 inflammasome activation in macrophages helps to protect mice against bacterial sepsis[144].

## 6. Therapeutic Implications

Given the growing evidence for the role of pyroptosis in liver injury and fibrosis, targeting liver pyroptosis represents a promising therapeutic option for the treatment of liver disease.

There are currently two major strategies for pharmacological inhibition of pyroptosis. One is to inhibit NLRP3 through regulatory pathways; for example, the NLRP3 inhibitor MCC950 effectively reduces liver injury and inflammation[129], type 1 interferon inhibits NLRP3 activation through the generation of nitric oxide or transcription of IL-10[145], and P2X7 inhibitors prevent ATP-mediated activation of NLRP3[146]. The other strategy is to inhibit downstream signaling pathways following NLRP3 activation; for instance, the effects of caspase-1 inhibitors[147], IL-1β inhibitor[148-150] and anti-IL-18[151] are being evaluated in clinical trials for some diseases.

GSDMD is the executive molecule in pyroptosis, but molecule targeting of GSDMD is rarely studied. There is only one study suggesting that necrosulfonamide, a known anti-necroptosis molecule, attenuates pyroptosis by directly binding gasdermin D and preventing pore formation in the membrane of septic mice[152]. The therapeutic value of this GSDMD inhibitor should be evaluated in liver diseases in the future.

Some herbal extracts and dietary components that have protective effects on the liver also inhibit pyroptosis. Silybin has long been known to inhibit NLRP3 inflammasome assembly through the nicotinamide adenine dinucleotide+/sirtuin 2(NAD+/SIRT2) pathway in mice with NAFLD[153]. Scutellarin is a natural flavonoid; it has been reported to inhibit NLRP3 inflammasome activation in macrophages and protect mice against bacterial sepsis by augmenting protein kinase A signaling[144]. Dihydroquercetin is the most abundant dihydroflavone found in onions. It ameliorates alcoholic liver steatosis by decreasing expression of NLRP3 and inhibiting IL-1β production and release[146]. Chlorogenic acid, a polyphenol found in coffee, fruits and vegetables, protects against carbon tetrachloride (CCl4)-induced acute liver injury probably through enhancing the anti-oxidant pathway and inhibiting NLRP3 inflammasome activation[154].

The details of current research regarding anti-pyroptosis in liver disease are listed in Table 2.

### Conclusion

In summary, pyroptosis is essential for the liver defense against pathogens and danger signals, but excessive pyroptosis promotes pathogenesis of various liver diseases. The pyroptotic process is complex, and its detailed molecular mechanism in liver disease requires further studies. For example, the role of pyroptosis has not been extensively studied in HBV- related diseases and in ACLF. A single study reveals the impact of renal DAMPs on the liver, yet the effects of liver DAMPs on the kidney remain unknown. The role of pyroptosis in the crosstalk between liver and the other organs requires further investigation.

There is no doubt that pyroptosis is a promising therapeutic target for inflammatory diseases. Inhibition of pyroptosis by blocking related molecules (e.g., NLPR3, caspases and IL-1) influences the progression of liver disease and provides a potential treatment approach for liver disease. However, as pyroptosis is an essential defensive line against pathogens, inhibition of pyroptosis may have a potential downside; for example, it may increase the risk of opportunistic infection. Therefore, safety is always an unavoidable concern. Much research is still required before the translation to clinical treatments. Further study of pyroptosis will contribute to the understanding of the mechanisms of hepatocellular injury and to the development of pharmaceutical inhibitors of pyroptosis.
